# A systematic review of antimicrobial resistance in *Neisseria gonorrhoeae* and *Mycoplasma genitalium* in sub-Saharan Africa

**DOI:** 10.1093/jac/dkac159

**Published:** 2022-05-17

**Authors:** Collins Iwuji, Diantha Pillay, Patience Shamu, Mercy Murire, Susan Nzenze, Laura-Ashleigh Cox, Saiqa Mullick

**Affiliations:** 1 Department of Global Health and Infection, Brighton and Sussex Medical School, University of Sussex, Brighton, UK; 2 Africa Health Research Institute, KwaZulu-Natal, South Africa; 3 Wits Reproductive Health and HIV Institute, Faculty of Health Sciences, University of Witwatersrand, Johannesburg, South Africa

## Abstract

**Objectives:**

Limited antimicrobial resistance (AMR) surveillance coupled with syndromic management of sexually transmitted infections (STIs) in sub-Saharan Africa (SSA) could be contributing to an increase in AMR in the region. This systematic review aimed to synthesize data on the prevalence of AMR in common STIs in SSA and identify some research gaps that exist.

**Methods:**

We searched three electronic databases for studies published between 1 January 2000 and 26 May 2020. We screened the titles and abstracts for studies that potentially contained data on AMR in SSA. Then we reviewed the full text of these studies to identify articles that reported data on the prevalence of AMR in *Neisseria gonorrhoeae, Chlamydia trachomatis, Trichomonas vaginalis* and *Mycoplasma genitalium* in SSA. We summarized the data using a narrative synthesis.

**Results:**

The 40 included studies reported on AMR data from 7961 *N*. *gonorrhoeae* isolates from 15 countries in SSA and 350 *M*. *genitalium* specimens from South Africa. All four SSA regions reported very high rates of ciprofloxacin, tetracycline and penicillin resistance in *N*. *gonorrhoeae*. Resistance to cefixime or ceftriaxone was observed in all regions except West Africa. Azithromycin resistance, recommended as part of dual therapy with an extended-spectrum cephalosporin for gonorrhoea, was reported in all the regions. Both macrolide and fluoroquinolone-associated resistance were reported in *M*. *genitalium* in South Africa. Studies investigating AMR in *C*. *trachomatis* and *T*. *vaginalis* were not identified.

**Conclusions:**

There is a need to strengthen AMR surveillance in SSA for prompt investigation and notification of drug resistance in STIs.

## Introduction

In 2016, the WHO estimated 376 million new infections globally with any of four sexually transmitted infections (STIs): chlamydia, gonorrhoea, syphilis and trichomoniasis. Daily, over 1 million new cases were recorded, with chlamydia as the commonest bacterial STI, although the majority of infections were attributed to trichomoniasis.^1^ Research into STIs is a relatively neglected area, despite the high global burden.

Low- and middle-income countries are disproportionately affected by STIs and have less developed laboratory infrastructure for the diagnosis and treatment of STIs. Furthermore, a lack of cheap point-of-care (POC) tests to diagnose STIs makes it difficult to implement a diagnostic approach. Consequently, symptomatic STIs are treated using the syndromic approach (presumptive treatment of those presenting with symptoms without laboratory diagnosis). However, syndromic treatment has poor specificity, resulting in overuse of antibiotics, which could be fuelling the increase in antimicrobial resistance (AMR).^2,3^ Furthermore, the majority of STIs are asymptomatic, especially in women,^4^ and typically go unnoticed and therefore are untreated. Hence, the syndromic approach creates both an over- and under-treatment paradox. Women bear the brunt of the complications of untreated STIs, including pelvic inflammatory disease, chronic pelvic pain, tubal factor infertility and pregnancy complications such as ectopic pregnancy, fetal or neonatal death, premature delivery and neonatal pneumonitis. STIs also increase HIV transmission and acquisition.^5–7^ *Mycoplasma genitalium*, a sexually transmitted pathogen that has been relatively neglected until recently, is also implicated in reproductive morbidity in women^8,9^ and HIV acquisition and transmission.^10,11^

The WHO Global Health Sector Strategy on STIs (2016–2021)^12^ envisions that, by 2030, rates of congenital syphilis will have reduced to <50 cases per 100000 live births in 80% of countries and the incidence of syphilis and gonorrhoea will have fallen by 90% globally between 2018 and 2030. However, the high global prevalence of *Neisseria gonorrhoeae* resistant to nearly all relevant antimicrobials previously and currently widely available for treatment, including sulphonamides, penicillins, tetracyclines, quinolones, early generation macrolides and cephalosporins threatens the achievement of these targets and underscores the critical need for new antimicrobial agents with activity against *N*. *gonorrhoeae*.^5^

Treatment guidelines should be informed by up-to-date, local and quality-assured surveillance efforts. More than 60 countries participate in the WHO Global Gonococcal Antimicrobial Surveillance Program (GASP), a worldwide laboratory network, which seeks to monitor resistance and provide data to inform treatment guidelines.^1^ One of the challenges of the programme is the variability in how countries undertake surveillance and susceptibility testing and how results are interpreted and reported to WHO.

Of the 47 countries in the WHO African region, only South Africa, Malawi, Ghana and Madagascar reported data to the WHO on monitoring outcome on gonorrhoea AMR in 2016, despite this region having one of the highest gonorrhoea burdens globally.^1^ Furthermore, there are limited data on AMR in STIs, including emerging STIs of clinical significance such as *M*. *genitalium* in the African region. This systematic review aims to undertake a narrative synthesis on the prevalence of AMR in *N. gonorrhoeae, Chlamydia trachomatis, M. genitalium* and *Trichomonas vaginalis* in sub-Saharan Africa (SSA) to identify some research gaps that may require further investigation. However, the review only identified studies that describe AMR in *N*. *gonorrhoeae* and *M*. *genitalium*.

## Methods

### Search strategy and study selection

This systematic review is reported according to the International Prospective Register of Systematic Reviews, with protocol number PROSPERO 2020 CRD42020197909. The search strategy was developed by four study authors (C.I., S.M., D.P. and P.S.) and was executed by P.S. on 26 May 2020 using the following search terms: (AMR OR antimicrobial resistance OR resistance) AND (Sexually transmitted infection OR sexually transmitted disease OR STI* OR STD* OR gonorrhoea OR chlamydia OR tri-chomon* OR mycoplasma genitalium) AND (Genital infections OR reproductive tract infection) AND (Sub-Saharan Africa). P.S. searched three electronic databases for published literature: PubMed, Web of Science and Embase. The search included studies published between 1 January 2000 and 26 May 2020. The identified articles were imported into Mendeley reference management software, which was accessible to study authors (S.M., C.I., D.P. and P.S.).

Using Mendeley we removed duplicates using the automated ‘Check for Duplicates’ function. We employed a three-step screening process, which commenced with an initial title screening, followed by an eligibility assessment of abstracts from the selected titles. Full-text articles of selected abstracts were then reviewed.

Screening was based on our inclusion criteria, which covered STI studies conducted in SSA amongst individuals aged 16 years and above, published in English, reporting on prevalence of AMR in the STIs of interest and published between 2000 and 2020. For multicountry studies, we only extracted data specific to SSA. We included studies that investigated AMR in more than 20 samples and were either cross-sectional, cohort, randomized controlled trials or surveillance in design. These were either prevalence studies or sets of laboratory-collected specimens. We excluded case reports, case–control studies, reviews, commentaries and editorials. We also did not search conference abstracts.

After the title screen, four study authors (C.I., S.M., D.P. and P.S.) independently screened the abstracts of identified articles for eligibility using a standardized data extraction table. Any discrepancies between identified articles were discussed amongst the four authors until an agreement was reached. We obtained the full text of the remaining articles and C.I., S.M. and D.P. independently screened them for eligibility and used the same process described above to resolve discrepancies. The final set of articles meeting our inclusion criteria were summarized by C.I., S.M. and D.P. in tables that had information on authors, study setting, study population, sampling year, study region, STI prevalence and AMR prevalence. Not all the studies reported on STI prevalence.

### Quality assessment

We used an adaptation of the Critical Appraisal Skills Programme quality assessment tool to assess the quality of the included studies.^13^ The criteria addressed the following questions: (i) Did the study address a clearly focused issue? (ii) Were the participants recruited in an acceptable way? and (iii) Was the outcome accurately measured to minimize bias? The potential responses selected for each of the questions were either ‘yes’, ‘no’, or ‘unclear’. Studies with ‘yes’ responses to all the questions were categorized as low risk of bias, whilst those in which any of the responses to the questions was either ‘no’ or ‘unclear’ were categorized as being at risk of bias (Table S1, available as Supplementary dataat *JAC* Online).

### Data synthesis and statistical analysis

We undertook a narrative synthesis of the included studies. The heterogeneity in study design and the different methods for performing antimicrobial susceptibility testing meant it was inappropriate to undertake statistical pooling of the data over time.

## Results

**Figure 1:**
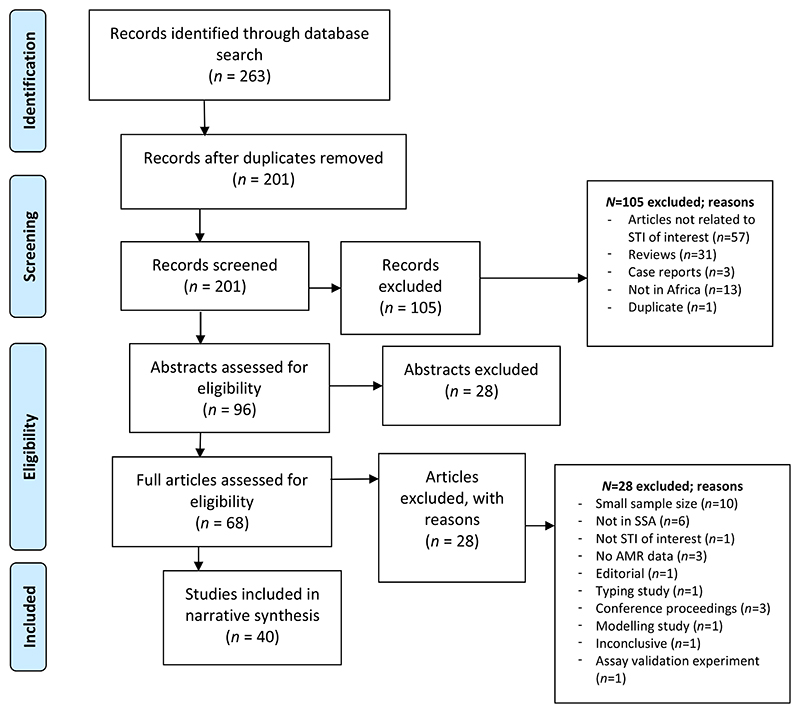
PRISMA diagram of the article selection procedures for articles published between 1 January 2000 and 26 May 2020. This figure appears in colour in the online version of *JAC* and in black and white in the print version of *JAC*.

Our initial search produced 263 articles, of which 62 were identified as duplicates. After screening the titles of the remaining 201 articles, we excluded another 105 articles whose titles were either not related to the subject of interest, or indicated they were reviews. We screened the abstracts of the remaining 96 articles and excluded a further 28 articles. Sixty-eight full-text articles were reviewed and 40 satisfied all inclusion criteria and contributed results to this systematic review (Figure 1).

These 40 studies included AMR data from 7961 *N*. *gonorrhoeae* isolates from 15 countries in SSA (Tables 1–4). The median sample size of *N*. *gonorrhoeae* isolates amongst the included studies was 139 (IQR 55–235; range 21–443). There were 350 *M. genitalium-*positive specimens examined for AMR and all were from South Africa. The median sample size of *M*. *genitalium* specimens amongst included studies was 43 (three studies; sample sizes 41, 43 and 266). The largest proportion of studies represented, 15/40 (38%), was from South Africa. Our review did not find any studies investigating AMR in *C*. *trachomatis* and *T*. *vaginalis*.

### Regional AMR patterns

#### Southern Africa

Twenty-one studies reported on AMR in the Southern Africa region;^14–34^ of which 15 were from South Africa,^15,17–22,26–32,34^ 3 from Zimbabwe,^23,25,33^ 2 from Malawi^16,24^ and 1 from Mozambique.^14^

Amongst the included studies, *N*. *gonorrhoeae* resistance to ciprofloxacin was first reported in 1999 in South Africa^34^ but a follow-up study 1 year later in the same province but a different city did not show any resistance.^18^ All the other South African studies reported ciprofloxacin resistance exceeding 5%.^15,19,22,28,29,31,32^ In two studies reporting a trend in ciprofloxacin resistance, there was an increase from 22% in 2003 to 42% in 2005^19^ in one study and an increase from 25% in 2008 to 69% in 2016 in the other study^32^ (Table 5). Two of the three studies from Zimbabwe examined ciprofloxacin resistance, with both studies reporting resistance of >5%.^23,33^ No ciprofloxacin resistance was observed in samples from 2005 in the only included study from Mozambique.^14^ Of the two included studies from Malawi, one reported ciprofloxacin resistance in *N*. *gonorrhoeae* of 6.1% from samples taken in 2000–01;^24^ however, a later study from a different region in Malawi did not observe ciprofloxacin resistance in *N*. *gonorrhoeae*.^16^

*N*. *gonorrhoeae* resistance to penicillin of 15% was first reported in South Africa in 1995, doubling to 30% in 1997 (*P* = 0.02) and remaining at about 30% until study end in 1999–2000.^27^ Over the same time period, high-level resistance to tetracycline increased from 3% in 1997 to 51% in 1998–99 (P < 0.001).^27^ This was corroborated by other studies in South Africa that examined *N*. *gonorrhoeae* resistance to penicil-lin^17,22,28,29,32^ and tetracycline.^17,18,22,26–29,32^ The study by Kularatne *et al*.^32^ showed a statistically significant increase in penicillin and tetracycline resistance from 2008 to 2016 (Table 5). The studies from Zimbabwe did not investigate penicillin and tetracycline resistance.^23,25,33^

*N*. *gonorrhoeae* resistance to extended-spectrum cephalosporins (ESCs) such as cefixime or ceftriaxone was low. Cefixime resistance of 1% was reported in Malawi in 2007^16^ and South Africa in 2014.^22^ Ceftriaxone-resistant gonorrhoea was not observed in nine studies that investigated this in samples from 1995 to 2017.^14,15,22,23,25,27,28,32,33^

Two South African studies examined azithromycin resistance.^22,32^ The study from KwaZulu-Natal reported azithromycin resistance of 68% in isolates from 2014,^22^ while the other in Johannesburg, which examined the trend of resistance from 2008 to 2017, only observed full resistance of 4.3% in *N*. *gonorrhoeae* from isolates analysed in 2008.^32^ However, the observed intermediate resistance to azithromycin decreased from 9.4% (22/233) in 2008 to 2.5% (3/122) in 2017, with no apparent MIC creep.^32^

Four studies reported on *N*. *gonorrhoeae* resistance to spectinomycin: two from Malawi^16,24^ and two from South Africa.^28,32^ In Malawi, spectinomycin resistance was reported as 11% and 0% in 2001^24^ and 2007, respectively.^16^ In South Africa, spectinomycin resistance was not found over a 10 year period in samples from 2008 to 2017, nor was resistance found in samples from 2003 in another study.^28,32^

*N*. *gonorrhoeae* resistance to gentamicin was only examined in two studies from Malawi. It was 15% in samples from a 2001 study^24^ and absent from samples taken in 2007.^16^

Only three studies from South Africa examined *M*. *genitalium* resistance to macrolides and/or fluoroquinolones.^20,21,30^ One study that examined specimens collected from 2007 to 2014 did not show any macrolide resistance in *M*. *genitalium*.^20^ QRDR mutations with known *M*. *genitalium*-associated fluoroquinolone resistance were not detected in the *gyrA* gene of DNA gyrase; however, one specimen (0.4%) contained a D87Y amino acid alteration in the *parC* gene that encodes the A subunit of topoisomerase IV, and has been linked to fluoroquinolone treatment failure.^20^ The study by Hay *et al*.^30^ reported macrolide resistance of 9.8% in sexually active women in samples from 2011 to 2012 whilst Ong *et al*.^21^ found no macrolide or fluoroquinolone resistance in samples taken from a cohort of HIV-positive women in the same period.

#### East Africa

Nine studies reported on AMR in the East Africa region,^35–43^ of which four were from Kenya,^37–39,43^ three from Ethiopia,^36,41,42^ one from Uganda^40^ and one from Rwanda.^35^

The earliest data were from Rwanda in 2000, which showed no *N*. *gonorrhoeae* resistance to ciprofloxacin.^35^ Prevalence of ciprofloxacin resistance was 11% overall in one Kenyan study on isolates from 2002 to 2009; no resistance was observed in isolates from 2002 to 2006, but a steady increase was observed afterwards from 9.5% in 2007 to 50% in 2009.^38^ All other studies that investigated ciprofloxacin resistance reported proportions ranging from 40.9% to 100%.^37,39–43^

The prevalence of *N*. *gonorrhoeae* resistance to penicillin was high in all included studies, ranging from 35.2% to 100%.^36–38,40–43^

Of the seven studies that reported on tetracycline resistance in *N*. gonorrhoeae,^36–38,40–43^ resistance was >90% in six of them.^37,38,40–43^

Of the four studies that reported on azithromycin resistance, one study in Uganda found a prevalence of 2.7% in isolates from 2009,^40^ with no resistance observed in the remaining three studies from Kenya.^37–39^

Spectinomycin resistance in gonorrhoea was not observed in the four studies that examined this.^35,37,38,40^

Three studies examined cefixime resistance; one study in Uganda found a prevalence of 1% in isolates from 2009,^40^ whilst two studies from Kenya did not find any resistance in isolates from 2002 to 2009^38^ and 2009 to 2010,^39^ respectively.

Eight studies examined ceftriaxone resistance in *N. gonorrhoeae,* five of which reported no resistance.^35,38–40,43^ The remaining three studies, which were from Ethiopia, reported a prevalence of 4.2% (year of isolates not specified),^36^ 27.8% in 2006–12^41^ and 48% in isolates from 2018.^42^

An Ethiopian study reported a prevalence of gentamicin resistance in *N*. *gonorrhoeae* of 14% in isolates from an unspecified year,^36^ while a Kenyan study reported a prevalence of 51% in isolates from 2020.^43^

#### West Africa

Six studies reported on AMR in the West Africa region: one from Ghana,^44^ one from Nigeria,^45^ two from Benin,^46,47^ one from Guinea Bissau^48^ and one from Cote d’Ivoire.^49^

The earliest documented ciprofloxacin resistance in *N*. *gonorrhoeae* was from a study in Guinea Bissau in isolates from 2006 to 2008, which reported a 10% prevalence.^48^ The Ghanaian study reported a prevalence of 81.8% in isolates from 2012 to 2015,^44^ and 62.3% in Nigeria in isolates from 2014 to 2016.^45^ No ciprofloxacin resistance was observed in isolates from 1998 to 1999 in Cotonou, Benin,^46^ but by 2015–17, this had increased to 75% in the same city.^47^

Amongst the included studies, penicillin resistance in *N*. *gonorrhoeae* was first documented in isolates from 1998 to 1999 in Benin, with a prevalence of 94.4%.^46^ High prevalence of resistance was reported in isolates from subsequent years in other countries in the region, ranging from 68% to 100% in five other studies.^44,45,47–49^

Tetracycline resistance in *N*. *gonorrhoeae* was equally high and was also first reported in Benin, with a prevalence of 99.3%.^46^ A later study from Benin^47^ and a study from Ghana reported a prevalence of 100%.^44^

Azithromycin resistance was examined in four studies. No resistance was found in isolates from Guinea Bissau^48^ and Benin.^47^ A study from Ghana reported a prevalence of 31.8% in isolates from 2012 to 2015^44^ while another study from Cote d’Ivoire reported a prevalence of 6.1% in isolates from 2017.^49^

Spectinomycin resistance in *N*. *gonorrhoeae* was not observed in four studies from Ghana,^44^ Benin,^46^ Guinea Bissau^48^ and Cote d’Ivoire.^49^

Resistance to the ESCs cefixime^47–49^ and ceftriaxone^46–49^ was not observed in the included studies.

#### Central Africa

The four included studies on AMR in Central Africa were from Cameroon;^50–53^ one of them was multisite and included samples from the Central African Republic.^51^ Three studies reported on ciprofloxacin resistance in *N*. *gonorrhoeae*; no resistance was observed in a study published in 2003, but the year of sampling was not specified for this study,^50^ while the other two studies reported on the prevalence trend in ciprofloxacin resistance.^52,53^ Tayimetha *et al*.^52^ reported a significant increase in ciprofloxacin resistance from 3.8% in 2009 to 50% in 2014, with resistance to penicillin and tetracycline remaining stably high during this period. Crucitti *et al*.^53^ reported a significant increase in ciprofloxacin resistance from 15% in 2012 to 79.5% in 2018 and tetracycline resistance remained stably high, whilst for penicillin resistance, although equally high during the period of observation, prevalence decreased significantly from 90.5% in 2016 to 68% in 2018.

Tayimetha *et al*.^52^ reported spectinomycin resistance of 2.6% but no trend data were given due to the small number of resistant isolates overall. Crucitti *et al*.^53^ reported an overall prevalence of 2% between 2012 and 2018, with no significant change in prevalence during this period. These two studies reported azithromycin resistance of 3.1%^52^ and 2.1%.^53^ Only the study by Crucitti *et al*.^53^ observed ceftriaxone resistance in *N. gonorrhoeae,* at 1.8% overall, with no significant difference in trend between 2012 and 2018.

### STI prevalence

Although AMR was the focus of this review, some of the included studies reported on STI prevalence.

#### Southern Africa

Of the 21 studies that reported on AMR prevalence in Southern Africa, 12 reported on STI prevalence: 6 in South Africa,^15,18,21,22,29,30^ 3 in Zimbabwe,^23,25,33^ 2 in Malawi^16,24^ and 1 in Mozambique.^14^

In South Africa, three studies evaluated men, all of which included those with urethritis,^15,18,22^ with one also examining men with genital ulcer syndrome or voluntary counselling and testing attendees.^15^ The prevalence of gonorrhoea in men with urethritis in the three studies ranged from 42.9% to 51%,^15,18,22^ with one of them reporting prevalence of 16%, 6.3% and 12.5% for chlamydia, *T*. *vaginalis* and *M. genitalium,* respectively.^15^ Three studies evaluated women^21,22,30^ but did not always state whether the women had symptoms or presented results according to the presence of symptoms. The prevalence of gonorrhoea was 2.3% and 10% in two studies,^21,22^ the prevalence of chlamydia was 5% in one study,^21^ the prevalence of *T*. *vaginalis* was 16.2% in one study^21^ and the prevalence of *M*. *genitalium* was 7.4% and 10% in two studies.^21,30^

Two of the three studies in Zimbabwe examined men with urethritis and reported gonorrhoea prevalence of 24.5% and 82.8%.^23,33^ One of the studies additionally reported prevalence of 11.7% for *C. trachomatis,* 1.6% for *T*. *vaginalis* and 4.7% for *M*. *genitalium*.^23^

The two Malawian studies were in men with urethritis and reported a prevalence of 80% or higher for gonorrhoea.^16,24^

#### East Africa

Of the nine studies that reported on AMR in East Africa, five reported on STI prevalence: three in Ethiopia^36,41,42^ and two in Kenya.^38,43^

In Ethiopia, two studies reported on gonorrhoea prevalence; one of them reported prevalence for only men, of 84.5%,^36^ and the other reported prevalence of 29% and 19% in men and women, respectively.^42^

Two studies in Kenya reported on STI prevalence; prevalence of gonorrhoea in men with urethritis in one study was 58.3%,^43^ while in the other study, gonorrhoea prevalence in men decreased from 3.8% in 2002 to 2.7% in 2009.^38^

No study in this region reported on the prevalence of *M*. *genitalium*.

#### West Africa

Of the six studies that reported on AMR in West Africa, five reported on STI prevalence: one in Nigeria,^45^ one in Ghana,^44^ one in Benin,^47^ one in Guinea Bissau^48^ and one in Cote d’Ivoire.^49^

The Nigerian study amongst MSM and transgender women reported gonorrhoea prevalence of 37.4%.^45^

The Ghanaian study amongst men with urethritis and women with vaginal discharge reported a gonorrhoea prevalence of 11% and 0.2% in men and women, respectively.^44^

The Benin study reported gonorrhoea prevalence of 16.4% amongst men with urethritis and women with vaginal discharge syndrome.^47^

The study from Guinea Bissau was amongst symptomatic women and reported prevalence of 0.6%, 8.4% and 4.2% for gonorrhoea, chlamydia and *T. vaginalis,* respectively.^48^

The Cote d’Ivoire study reported gonorrhoea prevalence of 2.5% (4.4% in males, 0.2% in females) amongst symptomatic and asymptomatic males and females attending sexually transmitted disease (STD) clinics.^49^

#### Central Africa

None of the four studies reporting on AMR in the Central Africa region reported on STI prevalence.

### Quality assessment of included studies

Our quality assessments of the included studies showed that all of the studies were clear about the focus of the research, but not all were clear about how participants were recruited. For example, some studies failed to indicate the dates study participants were recruited, making it difficult to compare their report of AMR with those of other studies.^36,50^ This was further complicated by the lag between identification of isolates and publication, variation in testing methodologies and difficulties in assessing the laboratory quality control procedures in the different studies. Twenty-five of the 40 included studies were assessed to be at low risk of bias (Tables 1–4and Table S1).

## Discussion

Our systematic review investigated AMR in *N*. *gonorrhoeae* and *M*. *genitalium* in SSA. The included studies comprised 7961 *N. gonor-rhoeae* isolates and 350 *M*. *genitalium*-positive specimens from 15 countries in this region.

We found a high prevalence of resistance to all antibiotics used for past and current treatment of gonorrhoea. Increasing ceftriaxone resistance was reported in Ethiopia,^36,41^ with low-level resistance (<5%) reported in Cameroon.^53^ While low-level cefixime resistance was observed in South Africa,^22^ Malawi^16^ and Uganda,^40^ resistance was not present in studies from West Africa^47–49^ and was not assessed in the four studies from Central Africa. Three of the four countries from the WHO African Region that contributed data to the 2016 WHO GASP (South Africa, Malawi, Ghana) reported resistance of <0.1% to ESCs, except in Madagascar where this was between 0.1% and 5%.1 The only Ghanaian study included in our review did not assess susceptibility to ESCs,^44^ whilst studies from Malawi^16^ and South Africa^22^ showed cefixime resistance of 1%, with no study demonstrating ceftriaxone resistance.

Azithromycin resistance in *N*. *gonorrhoeae* of <5% was reported in isolates from 2008 in Johannesburg, with subsequent isolates from the same centre showing no resistance in isolates from 2009 to 2017.^32^ However, an older study from KwaZulu-Natal reported high-level resistance in isolates from 2014.^22^ Azithromycin resistance of >5% was reported in Ghana^44^ and Cote d’Ivoire,^49^ whilst low-level resistance was observed in Uganda^40^ and Cameroon.^53^ The WHO GASP data on azithromycin resistance in Ghana and South Africa were consistent with our findings; however, this was not investigated in the Malawian study^16^ included in our review. High-level ciprofloxacin resistance was reported in the WHO GASP data from the four countries, consistent with our findings.

Resistance of *M*. *genitalium* to both fluoroquinolones and macrolides was only evaluated in South Africa, with resistance to both groups of antibiotics documented.^20,21,30^ Our search did not yield any published studies investigating resistance in chlamydia or trichomoniasis, hence we have focused the discussion on resistance in *N*. *gonorrhoeae* and *M*. *genitalium*.

In some of the countries in which ceftriaxone resistance in *N*. *gonorrhoeae* was not observed, there was a documented increase in the MIC of this antibiotic, suggesting that it may only be a matter of time before ceftriaxone treatment failure materializes. In order to prolong the therapeutic lifespan of ceftriaxone, this being the last option for first-line empirical chemotherapy for uncomplicated gonorrhoea, the WHO and other international guidelines recommend dual therapy for gonorrhoea with ceftriaxone and azithromycin.^54–56^ These guidelines vary in the preferred ESC, as well as in the doses of the regimens. The addition of azithromycin has the added benefit of treating possible chlamydia co-infection. Historically, the WHO has used a 5% threshold of AMR in *N*. *gonorrhoeae* to identify when empirical treatment with a particular antimicrobial agent is no longer ideal.^1^ High-level ceftriaxone resistance was reported in an Ethiopian study,^41^ although none of the included studies from Ethiopia investigated azithromycin resistance. However, in countries reporting azithromycin resistance ranging from 2.1% to 68%,^22,32,40,47,49,52,53^ it is reassuring that resistance to the ESC was low. The high level of resistance to azithromycin in KwaZulu-Natal, South Africa (68%)^22^ could be due to the use of this drug for other infections such as respiratory tract infec-tions;^22^ however, this pattern of resistance was not replicated in a more recent study in Johannesburg.^32^ This raises the question as to whether there could be geographical variation in azithromycin resistance in South Africa as none of the two studies is representative of the whole country. Kularatne *et al*.^32^ investigated participants attending a single public clinic in Johannesburg, whilst the Rambaran *et al*.^22^ study investigated participants from two public clinics in KwaZulu-Natal. Our systematic review cannot address the issue of a potential difference in the geography of azithromycin resistance in South Africa. Nevertheless, dual-therapy treatment failure of ceftriaxone and azithromycin is a real threat and was first reported in the UK in 2016.^57^ The WHO has indicated that such dual resistance in *N*. *gonorrhoeae* described in high-income countries may be a tip of the iceberg as the majority of gonorrhoea cases are in less-resourced countries where AMR surveillance is poor.^58^

The documented resistance to the macrolide azithromycin, as well as its inclusion for syndromic management of genital discharge and pelvic inflammatory disease syndromes, has implications for the treatment of *M*. *genitalium*. The contribution of *M*. *genitalium* to STI syndromes in SSA is understudied, although it is increasingly recognized as an important STI pathogen. In one South African study, the prevalence of *M*. *genitalium* was as high as that of other STIs.^21^ Only studies from South Africa reported on resistance in *M*. *genitalium*,^20,21,30^ with a prevalence of resistance to macrolides of up to 9.8% reported in samples prior to the introduction of azithromycin to syndromic treatment guidelines in South Africa in 2015.^30^ The increase in macrolide resistance in *M*. *genitalium* has been reported in countries where macrolides are frequently used, with resistance rates currently estimated at 30%–100% worldwide.^59,60^ This is a worrying trend as macrolides are first-line therapy for *M*. *genitalium* infections, with fluoroquinolones and tetracyclines being alternatives.

Although STI prevalence was not the focus of this systematic review, studies that reported on STI prevalence demonstrated a high prevalence of STIs amongst individuals with genital discharge, with prevalence being lower when STIs were assessed amongst general clinic attendees. The absence of diagnostic STI care, with reliance on syndromic management due to limited laboratory capacity and capability, poor antibiotic stewardship and high re-infection rates due to poor partner notification and poor recognition of treatment failure create the perfect condition for the emergence and spread of AMR in SSA.

A strength of our systematic review was being able to collate AMR data on gonorrhoea from 15 different countries, in addition to highlighting *M*. *genitalium* resistance to macrolides as an emerging public health problem. However, our review only captures AMR data from just under a third of countries in the region, suggesting there is still a lot of information lacking on AMR in the region.

This systematic review is subject to several limitations, which should be considered when interpreting the results. First, despite a systematic search of the aforementioned databases, we could have missed some important studies as we did not search the grey literature. Second, resistance data were reported across multiple studies. We excluded duplicate reports when we identified this, especially amongst studies reporting on trends, but it is possible some duplicate reports may have been missed. Third, there was a predominance of studies reporting on AMR in *N. go-norrhoeae*, with no studies identified reporting on AMR in chlamydia or trichomonas. Our search criteria may have failed to capture these studies. Fourth, we did not undertake a meta-analysis due to variation in the laboratory procedures to assess AMR and heterogeneity in the studied population. Fifth, there were no dates specified for when some samples were collected, making it difficult for us to compare these studies with other studies from the same region and across regions.

The WHO Global Action Plan on AMR describes AMR as a crisis, which poses a substantial threat to human health, that must be managed with the utmost urgency.^61^ This plan describes five objectives: (i) improving awareness and understanding of AMR; (ii) strengthening the knowledge and evidence base through surveillance and research; (iii) reducing new infections; (iv) improving antimicrobial stewardship; and (v) increasing investments in new medicines, diagnostic tools and vaccines. Achieving these objectives will require political commitment from African governments in order to provide the finance to develop the infrastructure necessary to tackleAMR. This will require developing capabilities and cap-acity for laboratory diagnosis of STIs in tandem with improvement in the early prevention, diagnosis, contact tracing, treatment and epidemiological surveillance of gonorrhoea cases.^62,63^

With AMR in *N*. *gonorrhoeae* to all currently recommended antimicrobials, research into new drugs is imperative. In this regard, zoliflodacin, the first in a new class of antibacterial agents called the spiropyrimidinetriones, inhibits bacterial type II topoisomerases^64^ and has shown promise in a Phase 2 study for the treatment of uncomplicated gonorrhoea.^65^ A large multicentre Phase 3 study is now in progress to evaluate the efficacy of zoliflodacin compared with dual treatment with ceftriaxone and azithromycin for the treatment of uncomplicated gonorrhoea.^66^ Other novel antimicrobial agents that have shown promising results are gepotidacin^67^ and solithromycin,^68^ with a few more in the pipeline.^63^

The US FDA has cleared a molecular POC diagnostic technology, which is easy to use and performs accurate chlamydia and gonorrhoea detection in 30 min.^69^ Other POC STI diagnostic technologies in various stages of development are also being evaluated.^5^ Currently, no commercial molecular POC STI technology allows for the detection of AMR, hence research to address this gap is required.^56^ Such POC STI diagnostic technology allows for the treatment of STIs during the same visit, thereby shortening the duration of infection and the likelihood of transmission to sexual partners. It also promotes good antibiotic stewardship by facilitating pathogen-based diagnosis and treatment. These technologies can complement the current syndromic management approach, as they do not require elaborate laboratory infrastructure, hence can be easily deployed in resource-constrained settings where the burden of STIs is greatest.

AMR is a global public health emergency, with drug-resistant *N*. *gonorrhoeae* being amongst the top five urgent antibiotic resistance threats to public health, according to the US CDC.^70^ The WHO names *N*. *gonorrhoeae* on its list of high-priority pathogens due to the emergence of resistance to ESCs and fluoroquinolones.^71^ The WHO GASP needs to be strengthened in many countries, especially in resource-constrained settings where the prevalence of gonorrhoea is high. Countries should receive technical support from the WHO to strengthen their AMR surveillance programme, in tandem with financial support from their governments to ensure that AMR to current antimicrobial agents is promptly detected and acted upon through update of treatment guidelines, if necessary. The introduction of enhanced surveillance that collects important epidemiological and clinical information such as age, same-sex partnerships, travel-associated sexual partnerships, or sentinel surveillance in specific groups, linked to microbiological or AMR data, might allow earlier identification of emerging resistance and risk factors that could allow more intensive follow-up and prevention interventions in groups at high risk of resistant gonorrhoea.^56,72^

Unless AMR in *N*. *gonorrhoeae* is tackled successfully through the development of new diagnostic and therapeutic agents, research into vaccine development, attention given to asymptomatic STIs, which account for the majority of STIs and are not addressed by syndromic management guidelines, and strengthened AMR surveillance to inform syndromic treatment guidelines, it will be challenging to achieve the WHO target of 90% reduction in gonorrhoea incidence by 2030.^1^ In this regard, it is of utmost importance to improve our understanding of the drivers of the emergence of AMR in *N*. *gonorrhoeae* and their mechanisms of resistance, which can provide an enhanced rationale for antimicrobial stewardship and management.^5^

## Supplementary Material

Supplementary Material

## Figures and Tables

**Table 1:** Summary of the included studies from Southern Africa

Authors	Study setting (sampling year)	Study population/ sample size	Study design	STI prevalence	Laboratory AMR methods	AMR prevalence	Risk of bias
Apalata *et al*.^14^	Maputo, Mozambique (2005)	270 patients were recruited; 116 with male urethritis syndrome and 154 with female discharge syndrome	Cross-sectional	NG was cultured from 40 (34.5%) men and 15 (9.7%) women	Agar dilution	NG: Ciprofloxacin: 0% Ceftriaxone: 0% Cefixime: 0% Kanamycin: 4/55 (7%) Tetracycline: 42/55 (77%) Spectinomycin: 0%	Low risk
Black et al.^15^	Johannesburg, South Africa (2004–06)	664 male participants with urethritis (*n* = 430), genital ulceration (*n* = 76) and HIV VCT clients (*n* = 158) attending the Esselen Street Clinic, Johannesburg	Prospective	NG: 285/664 (42.9%) CT: 106/664 (16.0%) MG: 83/664 (12.5%) TV: 42/664 (6.3%)	Etest susceptibility testing performed on 172 NG isolates	NG: Ciprofloxacin: 33/ 172 (19.2%) QRNG increased from 13.0% in the first year of the study to 26.3% in the second year (P = 0.03)	Low risk
Brown et al.^16^	Lilongwe, Malawi (2007)	126 men with urethritis attending the STD clinic at Kamuzu Central Hospital in Lilongwe	Cross-sectional	NG was cultured from 106 (84%) clients	Agar dilution susceptibility testing performed on 100 NG isolates	NG: Ceftriaxone: 0% Spectinomycin: 0% Gentamicin: 0% Cefixime: 1/100 (1%) Tetracycline: 77/100 (77%) Penicillin: 19/100 (19%) Ciprofloxacin: 0% Kanamycin: 0%	Low risk
Fayemiwo et al.^17^	South Africa (2008)	209 consecutive NG isolates, collected from men with urethral discharge	Cross-sectional	N/A	Etest	NG: Penicillin: 54/209 (25.8%) resistant and 87 (41.6%) reduced susceptibility Tetracycline: 157/ 209 (75.1%) and 37 (17.7%) reduced susceptibility	Low risk
Moodley et al.^18^	KwaZulu-Natal, South Africa (2000)	865 male patients presenting to the Prince Cyril Zulu Communicable Diseases Clinic in Durban with symptoms of urethritis (discharge and/or dysuria)	Cross-sectional	NG was isolated from 443 subjects: 177 (50%) in the 250 mg arm and 266 (52%) in the 500 mg arm	Agar dilution	NG: Ciprofloxacin: 0% Tetracycline: 284/ 443 (64%)	Low risk
Moodley *et al.^19^*	STI clinic, Durban, South Africa (2003, 2004, 2005)	139 NG isolates from patients with genital discharge; 259 NG isolates from patients with genital discharge; 248 NG isolates from men with urethritis	Cross-sectional	N/A	Agar dilution	NG/ciprofloxacin: 2003: 31/139 (22%) 2004: 62/259 (24%) 2005: 104/248 (42%)	At risk
Muller et *al.^20^*	STI surveillance programme & HIV outpatient clinic in Gauteng, South Africa (2007–14)	STI and HIV-positive patients; 266 MG-positive DNA extracts [126 men (45% HIV-positive) with median age of 28 years and 140 women (64% HIV-positive) with median age of 26 years]	Retrospective cross-sectional	N/A	23S rRNA gene mutation for macrolide resistance & mutations in QRDR of *gyrA* and *parC*	MG: Macrolide resistance: None Quinolone resistance: *parC*: 1/266 (0.4%) (D87Y amino acid alteration) *gyrA:* None	Low risk
Ong et *al.^21^*	Resident in Johannesburg, South Africa (2011–12)	Cervical specimens from 622 women with HIV, aged 25–50 years	Prospective cohort	Baseline: MG: 46 (7.4%) TV: 16.2% CT: 5.0% NG: 2.3% Follow up: MG 12/41 (29.3%)	23S rRNA gene mutation for macrolide resistance & mutations in QRDR of *gyrA* and *parC*	MG: Macrolide resistance: 23S rRNA: 0/43 (0%) Quinolone resistance: QRDR of gyrA: 0/26 (0%) QRDR of parC: 19/43 (44.2%)	Low risk
Rambaran *et al*.^22^	Two community health centres in Pietermaritzburg and Umlazi KwaZulu-Natal, South Africa (2014)	1220 male and female patients ≥18 years presenting with urethral/vaginal discharge	Cross-sectional	NG: 319/1220 (26%) Male 248/506 (49%) Female 71/714 (10%)	Agar dilution	NG: Azithromycin: 217/ 319 (68%) Penicillin: 193/319 (60%) Cefixime: 2/319 (1%) Ceftriaxone: 0% Ciprofloxacin: 223/319 (70%) Ofloxacin: 221/319 (69%) Tetracycline: 319/319 (100%) MDR (≥3 antimicrobials): 227/319 (71%)	Low risk
Takuva *et al*.^23^	Clinics in Harare, Zimbabwe (2010–11)	130 men ≥18 years presenting with urethral discharge	Cross-sectional	NG 106/130 (82.8%) CT 15/130 (11.7%) MG 6/130 (4.7%) TV 2/130 (1.6%)	Etest	NG: Ciprofloxacin: 4/66 (6.1%) Cefixime: 0% Ceftriaxone: 0% Kanamycin: 0%	Low risk
Zachariah *et al*.^24^	STI clinic, Thyolo Malawi (2000–01)	114 men with urethral discharge, median age 27 years	Cross-sectional	NG 91/114 (80%) CT 2/114 (2%)	Disc diffusion & Etest for gentamicin	NG: Gentamicin: 7/47 (15%) Penicillin: 43/47 (92%) Tetracycline: 38/47 (81%) Erythromycin: 23/47 (49%)Co-trimoxazole: 26/47 (55%) Spectinomycin: 5/47 (11%) Ciprofloxacin: 3/47 (6%)	Low risk
Mhondoro *et al.^25^*	Private microbiology lab in Harare Zimbabwe (2012–17)	23432 laboratory isolates from multiple sites examining multiple pathogens	Retrospective record review	NG 53/23432 (0.2%)	Disc diffusion	NG: Ceftriaxone: 0%	At risk
Moodley et al.^26^	STD clinic in KwaMsane, South Africa (1999)	204 NG isolates from men/women with urethral and genital discharge	Cross-sectional	N/A	Agar dilution	NG: 136/204 (67%) had MIC ≥16mg/L (cut-off for resistance). All were American variant of the tet(M) gene. Dutch tet(M) gene was not found	Low risk
Moodley et al.^27^	City health STD clinic in Durban, South Africa (1995–2000)	Patients presenting with genital discharge syndromes and diagnosed with NG (1995:61; 1997:198; 1998–99:98; 1999–2000:58). Overall: 415 NG isolates	Repeat cross-sectional	N/A	Agar dilution	NG:Ceftriaxone: 0% but increased MIC Spectinomycin: 0% but increased MIC Penicillin: 9/61 (15%) in 1995, increased to 60/198 (30%) in 1997 & remained at this level until 1999–2000 Tetracycline: 2/61 (3%) in 1997, increasing to 50/98 (51%) in 1998–99	Low risk
Moodley *et al*.^28^	STD clinic in Durban, South Africa (2003)	139 NG isolates from male patients with urethral discharge	Cross-sectional	N/A	Agar dilution	NG: Ciprofloxacin: 31/139 (22%) Tetracycline: 99/139 (71%) Ceftriaxone 0% Spectinomycin 0% Penicillin 41/139 (31%)	Low risk
Govender et αl.^29^	Port Elizabeth, South Africa (2003–04)	80 male patients with urethral discharge, dysuria or burning on micturition, and female patients with vaginal discharge attending clinics, aged 16–49 years	Called a cohort study by authors but actually a cross-sectional study	NG: 35/80 (43.8%)	Disc diffusion	NG: Ciprofloxacin: 21/35 (60%) resistant, 11/ 35 (31.4%) partially susceptible Doxycycline: 3/35 (8.6%) resistant Erythromycin: 28/35 (84%) resistant, 6/ 35 (17.1%) partially susceptible Penicillin: 17/35 (48.6%) resistant, 18/35 (51.4%) partially susceptible	At risk
Hay et al.^30^	South Africa (2011-12)	601 specimens from women (18–49 years) tested for MG	Cross-sectional	10.8% of women were infected with MG, either in the vagina or in the rectum. Vagina: 52/601 (8.7%; 95% CI 6.4–10.9) Rectum: 16/601 (2.7%; 95% CI 1.4–3.9)	MG: Macrolide resistance (mutations in 23S rRNA): 4/41 (9.8%); 2 resistant isolates from rectum and 2 from vagina	Low risk	
De Jongh et al.^31^	South Africa (2004–05)	141 NG isolates obtained from men presenting with urethritis to primary healthcare clinics and GPs	Cross-sectional	N/A	Agar dilution	NG: Ciprofloxacin: 10/141 (7.1%)	Low risk
Kularatne et al.^32^	Alexandra Health Centre, Johannesburg, South Africa (2008-17)	NG was cultured from genital discharge swab specimens obtained from consenting adult patients (see numbers of isolates tested in Table 5)	Longitudinal	N/A	Etest (cefixime, ceftriaxone, ciprofloxacin) or agar dilution (penicillin, tetracycline, azithromycin)	NG (numbers of isolates in Table 5): Penicillin: increase in resistance from 30% to 51% (trend *P<* 0.001) Tetracycline: from 75% to 83% (trend P = 0.008) Ciprofloxacin: from 25% to 69% (trend P<0.001) Spectinomycin: 0% & no MIC creep. ESCs: 0% & no MIC creep Azithromycin: 4.3% in 2008	Low risk
Latif et al.^33^	Zimbabwe (2015-16)	425 men ≥18 years of age, attending five sentinel clinics with urethral discharge	Cross-sectional	NG isolates: 104/425 (24.5%); 102 tested for resistance	Etests	NG: Ceftriaxone: 0% Cefixime: 0% Kanamycin: 2/102 (2%) Ciprofloxacin: 19/ 102 (18.6%) resistance overall, and ranged from 9.5% to 30.8% in the five sentinel sites; intermediate resistance 28/102 (27.5%)	Low risk
Moodley et al.^34^	South Africa (1999)	156 NG isolates from rural clinic and 204 NG isolates from urban clinics	Cross-sectional	N/A	Not specified	NG: Urban clinics: Ciprofloxacin: 0% Rural clinics: Ciprofloxacin: 3/156 (1.9%) Overall resistance: 3/360 (0.8%)	At risk

NG, *N*. *gonorrhoeae*; CT, C. *trachomatis*; MG, *M. genitalium;* TV, *T*. *vaginalis*; QRNG, quinolone-resistant NG; N/A, not available; VCT, voluntary counselling and testing.

**Table 2:** Summary of the included studies from Eastern Africa

Author	Study setting (sampling year)	Study population/ sample size	Study design	STI prevalence	Laboratory AMR methods	AMR prevalence	Risk of bias
Cehovin et al.^37^	Kenyan Medical Research Institute clinic in Mtwapa, Kenya (2010–15)	103 NG isolates from 73 patients (aged 18–49 years), including sex workers and MSM	Cohort study	N/A	WGS; disc diffusion for penicillin and tetracycline; Etest for ciprofloxacin, cefixime, penicillin, tetracycline, azithromycin and doxycycline	NG: 3 clusters identified; cluster 1 (30 isolates); cluster 2 (36 isolates) cluster 3 (11 isolates) & no cluster (26 isolates) Tetracycline (pTetM): 100/ 103 (97%) Doxycycline: all pTetM isolates resistant Ciprofloxacin: cluster 1 [28/30 (93%)], cluster 2 [28/36 (78%)], cluster 3 [10/11 (91%)] Penicillin: resistance in *penA* gene [cluster 1: 30/ 30 (100%); cluster 2: 30/ 36 (83%); cluster 3: 0/11 (0%)]; resistance in *ponA* gene [cluster 1: 30/30 (100%); cluster 2: 35/36 (97%); cluster 3: 11/11 (100%)] Azithromycin: 0% Spectinomycin: 0% Cefixime: 0%	At risk
Mehta et al.^38^	Kisumu, Kenya (2002-09)	331 NG diagnoses (culture & PCR) amongst 2784 men aged 18–24 years enrolled in a randomized trial of male circumcision to prevent HIV; 168 culture isolates were from 142 men	Cohort study	From February 2002 to July 2009, the prevalence of NG infection decreased from 3.8% in 2002 to 2.7% in 2009, representing 331 NG infections detected by PCR and/or culture	Agar dilution for 105 NG isolates + PCR to assess for QRNG in 61 isolates that were non-recoverable. Resistance assessed in total of 166/168 NG isolates	NG: Penicillin: 68/105 (65%) Tetracycline: 102/105 (97%) Spectinomycin: 0% Cefixime: 0% Ceftriaxone: 0% Azithromycin: 0% Ciprofloxacin (QRNG): 15/166 (9%) QRNG increased from 9.5% in 2007 to 50% in 2009 MIC creep over time: cefixime (P = 0.018), ceftriaxone (P<0.001) and azithromycin (P = 0.097)	Low risk
Nacht et al.^43^	UNIM Research & Training Centre clinic, Kisumu, Kenya (2018)	60 male patients attending routine STI clinics with history of discharge or dysuria	Cross-sectional	NG: 35/60 (58.3%)	Disc diffusion	Penicillin: 35/35 (100%) Doxycycline: 32/35 (91.4%) Tetracycline: 34/34 (100%) Ceftriaxone: 0/35 (0%) Ciprofloxacin: 34/34 (100%) Erythromycin: 0/30 (0%) Gentamicin: 18/35 (51.4%)	At risk
Tαdesse et *al*.^35^	Gondar Health Centre, Amhara region, Ethiopia (year not stated)	178 male patients presenting with urethral discharge (data analysed for 168); mean age 28 years	Cross-sectional	NG: 142/168 (84.5%)	Disc diffusion	NG: Chloramphenicol: 2/142 (1.4%) Erythromycin: 5/142 (3.5%) Ceftriaxone: 6/142 (4.2%) Gentamicin: 20/142 (14.1%) Kanamycin: 23/142 (16.2%) Tetracycline: 42/142 (29.6%) Carbenicillin: 52/142 (35.2%) Methicillin: 113/142 (79.6%) Ampicillin: 114/142 (71.3%) Penicillin: 121/142 (85.2%) Co-trimoxazole: 131/142 (92.3%)	At risk
Tibebu et al.^41^	Amhara Regional Health Research Lab, Bahir Dar, Northwest Ethiopia (2006–12)	Genital specimens from 352 male and female patients (mean age 28.1 years)	Retrospective cross-sectional	NG: 29/352 (8.2%)	Disc diffusion	NG: Ceftriaxone: 8/29 (27.8%) Ciprofloxacin: 12/29 (40.9%) Tetracycline: 27/29 (92.6%) Penicillin G: 28/29 (94.4%)	Low risk
Van Dyck et *et al*.^35^	PHC clinic, Kigali, Rwanda (1999–2000)	139 NG isolates from male adults with urethral syndromes	Cross-sectional	N/A	Agar dilution	NG: Ceftriaxone: 0/139 (0%) Ciprofloxacin: 0/139 (0%) Kanamycin: 0/139 (0%) Spectinomycin: 0/139 (0%) Co-trimoxazole: 30/139 (21.5%)	Low risk
Vandepitte *et al*.^40^	Kampala, Uganda (2008-09)	170 NG isolates from 148 female sex workers attending women’s clinic over an 18 month period; analysis done on 148 isolates	Prospective cohort	N/A	Etest	NG: Penicillin: 101/148 (68.2%) Cefixime: 1/148 (0.7%) Ceftriaxone: 0/148 (0.0%) Ciprofloxacin: 123/148 (83.1%) Spectinomycin: 0/148 (0.0%) Azithromycin: 4/148 (2.7%) Tetracycline: 144/148 (97.3%)	Low risk
Yeshanew *et al*.^42^	Gondar town, Northwest Ethiopia (2016)	120 patients (21 males + 99 females), mean age 27.8 years	Cross-sectional	NG: 25/120 (20.8%) Male 6/21 (29%) Female 19/99 (19%)	Disc diffusion	NG: Penicillin:19/25 (76%) Tetracycline: 25/25 (100%) Ciprofloxacin: 13/25 (52%) Ceftriaxone: 12/25 (48%) Cefotaxime: 7/25 (29%) Cefoxitin: 11/25 (44%) Clindamycin: 7/25 (28%) Ciprofloxacin + ceftriaxone: 11/25 (44%)	Low risk
Lagace-Wiens *et al*.^39^	Kenya (2009–10)	A total of 154 (82 female and 72 male) single isolates from high-risk clinic attendees from four cities	Cross-sectional	N/A	Disc diffusion or Etest, depending on study site	NG: Ciprofloxacin and/or norfloxacin: 82/154 (53.2%); 95% CI 45.3–61.8 Cefixime/ceftriaxone: 0% Azithromycin: 0%s	At risk

NG, *N*. *gonorrhoeae*; CT, *C*. *trachomatis*; MG, M. *genitalium*; TV, *T*. *vaginalis*; QRNG, quinolone-resistant NG; N/A, not available; pTetM, plasmid harbouring tet(M).

**Table 3:** Summary of the included studies from West Africa

Author	Study setting (sampling year)	Study population/ sample size	Study design	STI prevalence	Laboratory AMR methods	AMR prevalence	Risk of bias
Affolabi *et al*.^47^	Cotonou, Benin (2015-17)	146 samples tested from consecutive male & female patients presenting with urethritis, dysuria, cervicitis or vaginal discharge syndromes at two clinics	Cross-sectional	NG in 24/146 (16.4%) samples tested; (19 heterosexual men, 3 female sex workers and 2 MSM)	Not specified	NG: Azithromycin: 0/24 (0%) Cefixime: 0/24 (0%) Ceftriaxone: 0/24 (0%) Tetracycline: 24/24 (100%) Penicillin: 24/24 (100%) Ciprofloxacin: 18/24 (75%)	At risk
Attram *et al*.^44^	Accra, Sekondi and Takoradi (Ghana) (2012–15)	411 males and 579 females, presenting to five health facilities with urethral and vaginal symptoms	Cross-sectional	NG isolate obtained from 11% (n = 43) of males and 0.2% (n = 1) of females	Disc diffusion & confirmed by Etest	NG: Tetracycline: 44/44 (100%) Benzylpenicillin: 40/44 (90.9%) Ciprofloxacin: 36/44 (81.8%) Azithromycin: 14/44 (31.8%) Spectinomycin: 0%	At risk
Olsen *et al*.^48^	Sexual health and family planning clinics, Bissau, Guinea Bissau (2006–08)	711 women attending with urogenital problems; 27 men with NG were included	Prospective cohort	Women: CT 60 MG 30 NG 31 (27 from men)	Etest (described in another publication)	NG: Penicillin G: 21/31 (68%) Ampicillin: 21/31 (68%) Cefixime: 0 (0%) Ceftriaxone: 0 (0%) Cefuroxime: 0 (0%) Azithromycin: 0 (0%) Erythromycin: 2/31 (6%) Ciprofloxacin: 3/31 (10%) Spectinomycin: 0 (0%) Tetracycline: 23/31 (74%) Rifampicin: 4/31 (13%)	At risk
van Dyck *et al*.^46^	Cotonou, Benin (1998–99)	143 female sex workers with NG	Prospective	N/A	Agar dilution	NG: Ciprofloxacin: 0/143 (0%) Ceftriaxone: 0/143 (0%) Spectinomycin: 0/143 (0%) Co-trimoxazole: 3.5% Penicillin: 135/143 (94.4%) Tetracycline: 139/143 (99.3%)	Low risk
Yeo *et al*.^49^	Network of STD clinics in Abidjan and other parts of Cote d’Ivoire (2014–17)	9081 swab samples from 5065 men (median age 27 years) and 4016 women (median age 27 years)	Cross-sectional	NG: 230/9081 (2.5%) Male 222/5065 (4.4%) Female 8/4016 (0.2%) 212 NG isolates were available for susceptibility testing	Etest	NG: Ceftriaxone: 0/212 (0%) Cefixime: 0/212 (0%) Spectinomycin: 0/212 (0%) Gentamicin: 3/212 (1.4%) Azithromycin: 13/212 (6.1%) Ciprofloxacin: 133/212 (62.7%) Benzylpenicillin: 146/212 (68.9%) Tetracycline: 180/212 (84.9%)	At risk
Hardick *et al*.^45^	Nigeria (2014–16)	420 MSM tested for STI with 157 testing positive; 243 NG isolates in total as some MSM tested positive in ≥1 anatomical site; 183 isolates evaluated for resistance	Cross-sectional	NG: 157/420 (37.4%)	Genotyping	NG: Penicillin: 126/183 (68.8%) Ciprofloxacin: 114/183 (62.3%)	Low risk

NG, *N*. *gonorrhoeae*; CT, C. *trachomatis*; MG, *M*. *genitalium*; TV, T. *vaginalis*; N/A, not available.

**Table 4:** Summary of the included studies from Central Africa

Author	Study setting (sampling year)	Study population/ sample size	Study design	STI prevalence	Laboratory AMR methods	AMR prevalence	Risk of bias
Cao *et al*.^51^	Yaoundé, Cameroon and Bangui, Central African Republic (2004–05)	Outpatients with gonorrhoea attending the Pasteur Center of Cameroon in Yaoundé, Cameroon and health centres/ Pasteur Institute of Bangui in Central Africa Republic; 79 NG isolates from Cameroon, 30 NG isolates in Bangui	Cross-sectional	N/A	Agar dilution and disc diffusion	NG: Yaoundé: not done because all the strains were lost due to problems of electricity supply Bangui: Penicillin: 24/30 (86.7%) Spectinomycin: 0/30 (0%) Tetracycline: 30/30 (100%) Ceftriaxone: 0/30 (0%)	Low risk
Crucitti **et al*.^53^*	Yaoundé, Cameroon (2012–18)	449 NG isolates (296 from men and 153 from women)	Retrospective cohort	N/A	Disc diffusion (tetracycline, azithromycin & spectinomycin); Etest (ciprofloxacin, ceftriaxone & benzylpenicillin)	NG: Ciprofloxacin: 255/396 (64.4%) Benzylpenicillin: 311/391 (80.1%) Tetracycline: 240/411 (58.4%) Ceftriaxone: 7/390 (1.8%) Azithromycin: 9/428 (2.1%) Spectinomycin: 8/410 (2.0%) Resistance to ciprofloxacin increased significantly (P< 0.0001) from 15.0% (3/20) in 2012 to 79.5% (58/73) in 2018. Since 2016 a significant decrease (*P*= 0.002) in resistance to benzylpenicillin occurred while the resistance to tetracycline remained stable	At risk
Ndipetal.^50^	Central clinic, Tiko, Cameroon (year of sampling not specified)	32 NG isolates from a cohort of workers of both sexes at the Cameroon Development Corporation attending STI clinic with urethral/vaginal discharge	Prospective	N/A	Disc diffusion	NG: Penicillin: 32/32 (100%) Amoxicillin: 32/32 (100%) Spectinomycin: 32/32 (100%) Ciprofloxacin 0/32 (0%) Norfloxacin: 4/32 (1.4%) Ofloxacin: 4/32 (1.4%) Flumequine: 17/32 (51%)	At risk
Tayimetha *et al*.^52^	Hospitals and health centres in Yaoundé, Cameroon (2009–14)	129 men and 64 women with urethral/vaginal discharge diagnosed with NG (mean age 29.5 & 27 years, respectively)	Cross-sectional	N/A	Disc diffusion	NG: Benzylpenicillin: 180/193 (93.3%) Tetracycline: 113/193 (58.5%) Ciprofloxacin: 34/193 (17.6%) Chloramphenicol: 14/193 (7.3%) Azithromycin: 6/193 (3.1%) Spectinomycin: 5/193 (2.6%) Ceftriaxone: 0% Resistance to ciprofloxacin increased from 3.8% in 2009 to 50% in 2014 (P< 0.05)	At risk

NG, *N*. *gonorrhoeae*; CT, C. *trachomatis*; MG, *M*. *genitalium*; TV, T. *vaginalis*; N/A, not available.

**Table 5:** Number of *N*. *gonorrhoeae* isolates tested for susceptibility to various antimicrobials by calendar year, Johannesburg, 2008–17^32^

	Antimicrobials and antimicrobial susceptibility testing method	
Year	Cefixime, ceftriaxone, ciprofloxacin: Etest MIC (n)	Azithromycin, penicillin, tetracycline, spectinomycin: agar dilution MIC (n)
2008	338 (ceftriaxone and ciprofloxacin only)	233
2009	324	0
2010	316	0
2011	298	70
2012	294	31
2013	249	77
2014	235	93
2015	136	125
2016	128	113 (ciprofloxacin included)
2017	128 (cefixime and ceftriaxone only)	122 (azithromycin and spectinomycin only)
