# Supplementary material for: A systematic review of antimicrobial resistance in Neisseria gonorrhoeae and Mycoplasma genitalium in sub-Saharan Africa
Source: J Antimicrob Chemother. Author manuscript; Available in PMC 2022 Jul 31. (PMC9333409; doi:10.1093/jac/dkac159)
Supplement: Supplementary Material [file EMS146251-supplement-Supplementary_Material.docx]

**Supplementary data. Table S1. Quality assessment of included studies**

| **AMR Full Article Quality Assessment** | | | | |
| --- | --- | --- | --- | --- |
|  | Did the study address a clearly focused issue? | Were the participants recruited in an acceptable way? | Was the outcome accurately measured to minimise bias? | Risk of Bias |
|  | *A question can be ‘focused’ in terms of the population studied or the outcomes considered* | *Look for selection bias which might compromise the generalisability of the findings: • was the cohort representative of a defined population • was there something special about the cohort • was everybody included who should have been* | *measurement or classification bias: • use of subjective or objective measurements •are measures validated • has a reliable system been established for detecting all the cases (for measuring disease occurrence) • measurement methods similar in the different groups* | At Risk or Low Risk |
| Affolabi et al., 2018 | Yes | Yes | Unclear | At risk |
| Apalata et al., 2009 | Yes | Yes | Yes | Low risk |
| Attram et al., 2019 | Yes | Unclear | Yes | At risk |
| Black et al., 2008 | Yes | Yes | Yes | Low risk |
| Brown B et al., 2010 | Yes | Yes | Yes | Low risk |
| Cao V et al., 2008 | Yes | Yes | Yes | Low risk |
| Cehovin et al., 2018 | Yes | Unclear | Yes | At risk |
| Crucitti et al., 2020 | Yes | Unclear | Yes | At risk |
| Fayemiwo et al., 2011 | Yes | Yes | Yes | Low risk |
| Kularatne R et al., 2018 | Yes | Yes | Yes | Low risk |
| Lagace-Wiens P et al., 2012 | Yes | Unclear | Yes | At risk |
| Latif A et al., 2017 | Yes | Yes | Yes | Low risk |
| Mehta S et al., 2011 | Yes | Yes | Yes | Low risk |
| Moodley P et al., Lancet 2001 | Yes | Unclear | Yes | At risk |
| Moodley P et al., JAC 2001 Tet Resistance | Yes | Yes | Yes | Low risk |
| Moodley P et al., JAC 2001 AMR in NG | Yes | Yes | Yes | Low risk |
| Moodley P et al., IJAA 2002 low dose Cipro | Yes | Yes | Yes | Low risk |
| Rambaran S et al., 2019 | Yes | Yes | Yes | Low risk |
| Tadesse A et al., 2001 | Yes | Unclear | Yes | At risk |
| Govender S *et al.,* 2006 | Yes | Unclear | Yes | At risk |
| Hardick *et al.,* 2018 | Yes | Yes | Yes | Low risk |
| Hay *et al.,* 2015 | Yes | Yes | Yes | Low risk |
| Jongh *et al.,* 2007 | Yes | Yes | Yes | Low risk |
| Mhondoro *et al.,* 2019 | Yes | Unclear | Yes | At risk |
| Moodley *et al.,* 2006 | Yes | Yes | Yes | Low risk |
| Moodley & Sturm; 2005 | Yes | Unclear | Unclear | At risk |
| Muller *et al.,* 2019 | Yes | Yes | Yes | Low risk |
| Nacht *et al.,* 2020 | Yes | No | Yes | At risk |
| Ndip *et al.,* 2003 | Yes | Unclear | Yes | At risk |
| Olsen *et al.,* 2012 | Yes | Unclear | Yes | At risk |
| Ong *et al.,* 2020 | Yes | Yes | Yes | Low risk |
| Takuva *et al.*, 2014 | Yes | Yes | Yes | Low risk |
| Tayimetha *et al*., 2018 | Yes | Yes | Yes | At risk |
| Tibebu *et al*., 2013 | Yes | Yes | Yes | Low risk |
| Tsai *et al.*, 2013 | Yes | Yes | Yes | Low risk |
| van Dyck *et al*., Int. J STD & AIDS 2001 | Yes | Yes | Yes | Low risk |
| Vandepitte *et al.*, 2014 | Yes | Yes | Yes | Low risk |
| Yeo *et al.,* 2019 | Yes | Unclear | Yes | At risk |
| Yeshanew and Gerenew et al., 2018 | Yes | Yes | Yes | Low risk |
| Zachariah *et al.,*2003 | Yes | Yes | Yes | Low risk |
